# Foot and Ankle Disorders in Nurses Exposed to Prolonged Standing Environments: A Scoping Review

**DOI:** 10.1177/21650799221137646

**Published:** 2023-01-13

**Authors:** Rafael A. Bernardes, Sílvia Caldeira, Pedro Parreira, Liliana B. Sousa, João Apóstolo, Inês F. Almeida, Paulo Santos-Costa, Minna Stolt, Arménio Guardado Cruz

**Affiliations:** 1 Nursing School of Coimbra (ESEnfC), Coimbra; 2 Universidade Católica Portuguesa, Lisbon; 3 University of Turku, Turku

**Keywords:** foot diseases, ankle injuries, nurses, standing position, occupational health

## Abstract

**Background:**

Prolonged standing environments constitute an occupational risk factor for nurses, particularly for developing foot and ankle disorders. The definitions and potential relationship to hours spent walking or standing are poorly understood. This scoping review aimed to synthesize the main disorders found on nurses’ ankles and feet, their prevalence, the influence of hours spent walking or standing, and gender differences.

**Methods:**

This review followed a previously published protocol. Primary and secondary studies were retrieved from relevant databases from December 2020 to March 2021. Potential articles were collated to Mendeley, and two independent reviewers assessed the title and abstracts. Studies meeting inclusion criteria were included. Two researchers retrieved and reviewed the full text of these studies independently. A predetermined extraction tool was used to retrieve relevant data, summarized in a tabular and narrative format.

**Findings:**

The most common disorder was pain, followed by numbness, burning feet, bunions, structural deformities, and calluses. Prevalence differed among studies, depending on settings and specific local policies. *Discussion:* Various foot and ankle disorders and related variables have been found, with clear gaps that may be addressed in the future.

**Conclusion/Applications to Practice::**

Few studies have focused on nurses’ foot and ankle disorders. Mapping signs and symptoms may contribute to the future development of preventive interventions for nurses’ workplaces.

## Background

According to a recent report by the European Agency for Safety and Health at Work (EU-OSHA, 2021) entitled, *Prolonged constrained standing at work—Health effects and good practice advice*, one in five workers in the European Union (EU; 20%) spend most of their working time standing, 60% identify musculoskeletal disorder (MSD) as the most severe issue, and 29% report muscular pain in the lower limbs in the past 12 months. Moreover, the International Labour Organization (ILO, 2013) considers MSD a significant public health concern in Europe, being the most frequent work-related health disorder.

In general, prolonged walking and standing contexts are associated with decreased blood circulation to the lower extremities, reducing nutrient supply to muscles, thus leading to fatigue and pain (EU-OSHA, 2019; Peddie et al., 2021). The manifestations of MSDs can be divided into specific (clear clinical features) or nonspecific disorders such as pain without evidence of clear specific disorders (Krishnan et al., 2021).

Many occupations require prolonged daily standing, such as food service, factory, retail environments (Anderson et al., 2018), and the healthcare professions, specifically nursing. While performing interventions in static positions or walking long distances throughout the day, nurses are exposed to consequences associated with prolonged walking and standing, predisposing their lower extremities to stress, especially in the ankle and foot region (Stolt et al., 2018). Getie et al. (2021) state that 24% of nurses were absent from work in the past 12 months due to foot and ankle pain, which was the second reason for absenteeism among healthcare professionals.

According to Getie et al. (2021), foot and ankle pain is a sensory and emotional experience caused by inflammatory and degenerative damage to any region distal to the tibia or fibula, including bones, joints, muscles, nerves, skin, and vascular structures. In addition, constant exposure to prolonged standing has a 1.7-fold increase in the risk of foot pain (Anderson et al., 2018).

The existing evidence on foot and ankle disorders among nurses is particularly challenging for research as it is dispersed and very heterogeneous, with a clear dependence on the type of occupational context. Regarding the prevalence of pain, values range from 21.2% in Portugal (Moura et al., 2019), 23% in Japan (Tojo et al., 2018), 42% in Poland (Rypicz et al., 2020), to as much as 55.3% in Australia (Reed et al., 2014).

Although it is an area identified as a priority to improve the quality of life among nurses (Ou et al., 2021), research focusing on nurses’ feet is scarce (Stolt et al., 2017), and a preliminary search of the literature has revealed that no reviews have been conducted or are underway about this topic. Furthermore, few studies focus on the relationship between prolonged standing, signs and symptoms of foot and ankle disorders, hours spent walking or in static positions, and gender-based differences.

Therefore, our objective was to identify the main foot and ankle disorders among nurses, their prevalence, the influence of hours spent walking or standing, and gender differences.

## Methods

This scoping review was guided by the Preferred Reporting Items for Systematic Reviews and Meta-Analyses Extension for Scoping Reviews (PRISMA-ScR; Tricco et al., 2018) and by the protocol with the respective methodology, which has been published previously (Bernardes et al., 2021).

### Inclusion and Exclusion Criteria

The inclusion criteria were defined through participants, concept, and context. Regarding participants, this review considered studies that include only nursing professionals and midwives. The concept of the study refers to “foot and ankle disorders,” whether of a musculoskeletal, neurological, or other nature. As for context, this review considered studies concerning standing environments in acute hospital settings. As such, studies with nurses who usually work in a stationary environment for most of the time (e.g., clinical appointments) were excluded.

### Data Extraction

The scoping review was conducted on a total of 12 databases, search engines, and research repositories: Medical Literature Analysis and Retrieval System Online (MEDLINE), Cumulative Index to Nursing and Allied Health Literature (CINAHL), Latindex, Scientific Electronic Library Online (SciELO), Web of Sciences, Cochrane Database of Systematic Reviews, Joanna Briggs Institute Clinical Online Network of Evidence for Care and Therapeutics (JBI Connect Plus), PROSPERO, and, for unpublished studies and gray literature, Google Scholar, Open Gray, Directory of Open Access Repositories (OpenDOAR), and ProQuest Dissertation and Theses Global.

An initial limited search of MEDLINE (through PubMed) was conducted with pilot keywords related to the participants, the concept, and the context, leading to the development of a full search strategy, using the index terms in titles and abstracts. The next step included an extensive search of the remaining databases. Finally, the reference lists of all included papers were searched manually for additional studies of interest.

The search strategy used for MEDLINE (through PubMed) was further adapted for the remaining databases, taking into consideration specific thesaurus, and can be found in Supplemental Table 1. Relevant filters applied to all databases were limited to Portuguese, English, French, and Spanish results and studies published from the earliest point until March 2021.

**Table 1.** Summary of Retrieved Studies (*N* = 38) Regarding Foot/Ankle Disorders in Nursing Standing Environments

Author(s), years, country	Design	Population and sample size	Foot/ankle disorders and signs/symptoms	12-month prevalence	Relation to other body parts	Walking/standing hours	Gender specifications
Schaefer (1982), United States	Exploratory study	Nurses (sample size not reported)	Cold feet; numbness, tingling, or burning of the toes/foot; fatigue; leg muscle cramps; or pain				
Sabine, (1999), United Kingdom	Narrative study	Nurses (sample size not reported)	Calluses and corns, hyperhidrosis, fungal infections, contact dermatitis, onychomycosis, ingrown toenail, verrucae/warts, foot strain, or metatarsalgia or generalized forefoot pain				
Smith et al. (2002), Taiwan	Observational study	75 nurses	Fungal infections				
Kee and Seo, (2007), Korea	Observational study	Not reported	Pain, ache, numbness, burning, swelling, or discomfort				
Reed (2007), United States	Observational study	304 nurses	Bunions, curled toes, flat feet, high arch, callouses/corns, heel spurs, or pain	54%	Third most prevalent, after lower back (69.9%) and neck problems (66%)		
Nealy et al. (2012), United States	Exploratory study	460 nurses	Pain, hallux rigidis, bunion, hammer toes, ankle sprain, or Achilles tendonitis				
Attar (2014), Saudi Arabia	Cross-sectional study	200 nurses	Pain, numbness, tingling, aching, stiffness, or burning feet				
Li (2017), United States	Mixed-methods study	496 nurses	Arthritis, multiple ankle sprains, plantar fasciitis, fracture, anterior tibial tendonitis, stress fracture, tendonitis, tarsal tendon, bunion, bone spurs, bipartite sesamoid, Morton’s neuroma, hammer toe, avascular necrosis of the metacarpals, peripheral neuropathy, metatarsalgia, gout, extra metatarsals bone, osteoarthritis, hallux rigidis, Achilles tear, Achilles tendonitis, flat foot, posterior tibial tendonitis, or torn ligament				
Stolt et al. (2017), Finland	Cross-sectional study	411 nurses	(Skin health) Dry skin, corns or calluses, cold feet, leg cramps, sweating feet, fissures in the heels, edema, burning feet, skin breaks or maceration between toes, verrucae (nail health), thickened toenail, color changes in the nails, ingrown toenail, fungal infection of the nails, (foot structure) low foot arch, hallux valgus, Taylor bunion, hammer toes, or high foot arch				
Moura et al. (2019), Portugal	Correlational study	260 nurses	Pain in foot/ankle	21.2%	First lowest prevalent, followed by elbows (14.6%)		
Dulina et al., (2019), Slovakia	Observational study	468 nurses	Pain incidence in ankles and feet				
Amin et al., (2020), Malaysia	Cross-sectional study	550 nurses	Pain, numbness, tingling, aching, stiffness, or burning	47.2%	Most prevalent, followed by the upper back (40.7%) and shoulders (36.9%)		
Li, Sommerich, Chipps, Lavender and Stasny (2020), United States	Literature review	766 nurses	Bunions, corns and calluses, plantar warts, plantar fasciitis, flat foot, or high arch				
Stolt et al. (2016), Finland	Narrative review	35 studies	Foot pain	Ranges between 3.2% and 100%.	Most prevalent		Gender seems to be a significant factor for MSDs, particularly for ankle/foot symptoms. Female nurses have more knee pain, whereas male nurses have ankle pain more often than female nurses.
Choobineh et al. (2006), Iran	Cross-sectional study	641 nurses	Ache, pain, discomfort, or numbness	52.1%	Most prevalent		
Almhdawi et al. (2020), Jordan	Observational study	597 nurses		28.5%	First most prevalent, following the lower back (77.4%) and knees (37.5%)	44.2% reported sustaining the same position for an extended period of one to three times/day	
Nur et al. (2016), Malaysia	Cross-sectional study	376 nurses		47.2%	The second most prevalent following the neck (48.9%).		
Choobineh et al. (2010), Iran	Cross-sectional study	375 nurses		59%	Second most prevalent, following lower back (60.6%)		Women significantly more affected than men (*p* < .05)
Reed et al. (2014), Australia	Cross-sectional study	304 nurses		55.3%	Third most prevalent following		
Serranheira et al. (2012), Portugal	Cross-sectional study	2,140 nurses		24.83%	First lowest prevalent,followed by knees(21.74%), thighs(10.51%), and elbows(7.49%).		
Gurgueira et al. (2003), Brazil	Cross-sectional study	105 nurses		14.3%	First lowest prevalent, followed by elbows (7.6%)		
Bazazan et al. (2019), Iran	Cross-sectional study	380 nurses		39%	The second lowest prevalent, followed by elbows (29%).		
Nguyen et al. (2020), Vietnam	Cross-sectional study	1,179 nurses		7.2% (men) 8.8% (women)	Third lowest prevalent, followed by elbows/forearm (5.9%) and hip/thigh (3.2%)		The prevalence of MSD at each anatomical site in women was higher than in men, except for ankle/foot.
Santos et al. (2017), Brazil	Cross-sectional study	29 nurses		55.2%	Fourth lowest prevalent, followed by wrists/hands (51.7%), thighs (34.5%), knees (34.5%), and elbows (24.1%).		
Hou and Shiao (2006), Taiwan	Cross-sectional study	5,271 nurses		14.4%	Lowest prevalent		
Kołcz et al. (2019), Poland	Observational study	37 nurses		16%	Lowest prevalent	41% of the nurses spend more than 8 hours in standing positions; 59% from 5 to 8 hours in this position.	
Tariq et al. (2018), Pakistan	Cross-sectional study	369 nurses		35.5%	^a^		
Sorour and El-Maksoud (2012), Egypt	Cross-sectional study	58 nurses		44.8%	^a^		
Raithatha and Mishra (2016), India	Observational study	296 nurses		27%	^a^		
Yang et al. (2018), China	Cross-sectional study	679 nurses		31.5%	^a^		More prevalence in women
Li, Sommerich and Chipps, (2020), United States	Mixed-methods study	20 nurses				Medical/surgical units: 53.6% of shift standing, 14.7% of shift walking, and 68.3% of the shift being on their feet (includes time standing and walking); ICU units: 56.3% of the shift standing, 14.05% of shift walking, and 70.4% of the shift being on their feet (includes time standing and walking)	
Júnior et al., (2019), Brazil	Cross-sectional study	143 nurses					
Magnago et al. (2008), Brazil	Literature review	18 documents	Pain				
Baty and Stubbs (1987), United Kingdom	Observational study	52 nurses	Pain			Standing accounted for 21.8% of shift time (56.6% in the early shift; 53.8% in the late shift). Walking accounted for 53.7% of shift time (18.6% in early shift; 21.0% in late shift).	
Antochevis-de-Oliveira et al. (2017), Brazil	Cross-sectional study	149 nursing students	Pain	41.6%	Fifth most prevalent, following the upper back (73.8%), lower back (67.1%), shoulders (52.3%), and neck (42.3%).		Women have more pain.
Gonçalves et al. (2001), Brazil	Cross-sectional study	29 nurses	Pain			Day shift nurses spent more than 70% of the time standing (8.4 hours). Night shift workers spent about half the shift (51%) standing (6.12 hours).	
Omidi et al. (2018), Iran	Cross-sectional study	120 nurses	Pain				Higher rates of discomfort and severity of discomfort in women than in men
Pinar (2010), Turkey	Cross-sectional study	2,400 nurses	Pain, numbness, tingling, aching, stiffness, or burning				

*Note*. MSD = musculoskeletal disorder; ICU = intensive care unit.

a Prevalence values appear among others, and the classification would be either redundant or irrelevant.

Databases were searched for original articles reporting primary research and secondary research, such as literature reviews and other relevant documents. Gray literature was also included for a more comprehensive review and to reduce bias in the findings (Paez, 2017). The first database to be searched was MEDLINE (through PubMed) in December 2020, and the last database to be searched was CINAHL Complete (through EBSCOhost) in March 2021. All documents were first screened by two independent reviewers, considering the title and abstract, and the second stage was based on the full-text reading. When the title or abstract had insufficient information to assess inclusion, the reviewers performed a full-text analysis. Final data were extracted from a tool previously developed (Bernardes et al., 2021). Any disagreements between reviewers were solved by discussion or with a third reviewer. Data have been organized using tables, as defined in the protocol.

Mendeley® reference manager (Elsevier®) was used to collate studies and perform the screening and selection.

## Results

**Figure 1.**
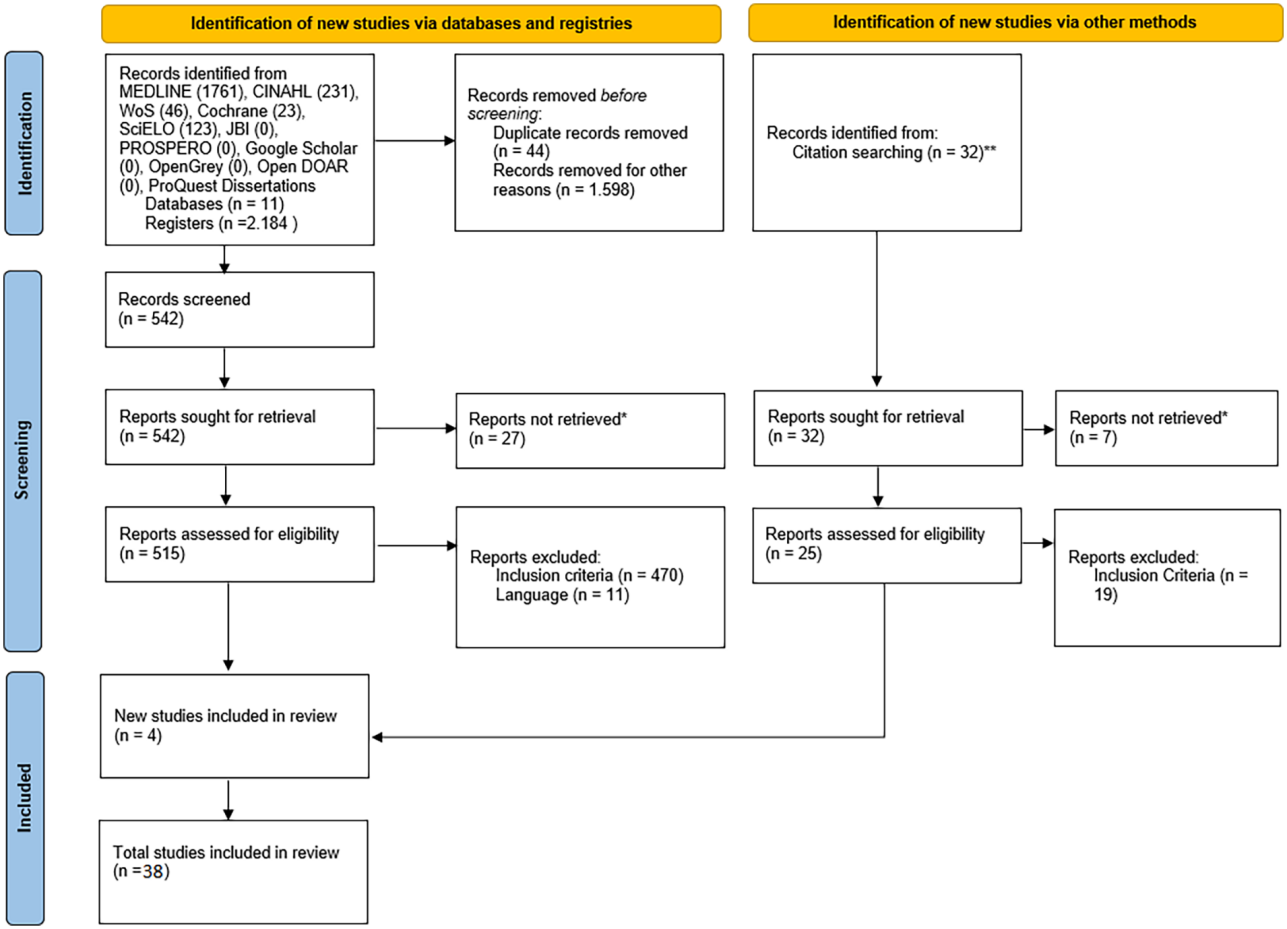
PRISMA-ScR flowchart. *Note*. PRISMA-ScR = Preferred Reporting Items for Systematic Reviews and Meta-Analyses Extension for Scoping Reviews. ^a^ Full texts were not available and original authors were contacted but gave no feedback. ^b^ Manual searching.

A total of 2,184 results were retrieved from the selected databases, search engines, and repositories (Figure 1). After identifying relevant studies and excluding those that did not meet the criteria or were duplicated, 515 studies were screened for eligibility. Following this step, and after a manual identification from citation searching, a total of 39 studies were included in the review.

The studies (*N* = 38; Table 1) were published between 1982 and 2020, and were developed in several countries, namely, Brazil (*n* = 6), United States (*n* = 6), Iran (*n* = 4), Malaysia (*n* = 2), Portugal (*n* = 2), Finland (*n* = 2), United Kingdom (*n* = 2), Taiwan (*n* = 2), Australia (*n* = 1), Pakistan (*n* = 1), China (*n* = 1), Turkey (*n* = 1), Egypt (*n* = 1), Poland (*n* = 1), Saudi Arabia (*n* = 1), Vietnam (*n* = 1), Korea (*n* =1), Jordan (*n* = 1), India (*n* = 1), and Slovak Republic (*n* = 1). The most common study designs were cross-sectional (*n* = 22; 57.89%). As for gray literature, two doctoral theses were retrieved (Li, 2017; Reed, 2007).

### Foot and Ankle Disorders and Relevant Signs and Symptoms

The most common disorder reported in the literature was pain, reported explicitly in 19 studies (Table 2), followed by numbness, burning feet, bunions/hallux valgus/bone spurs, structural deformities—low or high foot arches and flat feet, fungal infections, and calluses/corns.

**Table 2.** Most Frequent Foot Disorders and Respective Signs and Symptoms

Disorders	Signs and symptoms	Studies
Calluses, corns	• Areas exposed to intense/extreme pressure and friction forces; • Thickened, inflexible skin, often on the sole; • Symptom of a bone deformity, a particular style of walking, or inappropriate footwear.	Sabine (1999), Reed (2007), Stolt et al. (2017), and Li, Sommerich, Chipps, Lavender and Stasny (2020).
Hyperhidrosis	• Maceration.	Sabine (1999) and Stolt et al. (2017).
Fungal infections	• Presence of moisture and warmth; • Itchy skin; • Skin flakes, peels, or cracks.	Schaefer (1982), Sabine (1999), Smith et al. (2002), and Stolt et al. (2017).
Contact dermatitis	• Dry and shiny inflamed skin; • Skin cracks; • Demarcation along the line of the shoe.	Sabine (1999) and Stolt et al. (2017).
Onychomycosis	• Thickened nails; • Brittle and yellowish-brown toenails; • Color changes.	Schaefer (1982), Sabine (1999), and Stolt et al. (2017).
Ingrown toenail	• Inappropriate nail cutting, tight-fitting shoes, pressure on the skin around the nails, and/or moist skin; • Swelling with concomitant infection and pain.	Sabine (1999) and Stolt et al. (2017).
Verrucae, warts	• Common on the soles; • Darkish brown, rough, and crumbly surface; • Hard skin.	Sabine (1999), Stolt et al. (2017), and Li, Sommerich, Chipps, Lavender and Stasny (2020).
Foot/ankle strain and sprain	• General fatigue/pain of the lower limb; • Aching/tenderness in the midfoot, hindfoot, and leg muscles.	Sabine (1999) and Li (2017).
Pain, metatarsalgia, and generalized forefoot pain and discomfort	• Pain (generalized; local at the ball of the foot); • Inflammation; • Discomfort.	Schaefer (1982), Sabine (1999), Gurgueira et al. (2003), Hou and Shiao (2006), Reed (2007), Kee and Seo (2007), Magnago et al. (2008), Choobineh et al. (2010), Sorour and El-Maksoud (2012), Nealy et al. (2012), Stolt et al. (2016), Raithatha and Mishra (2016), Antochevis-de-Oliveira et al. (2017), Santos et al. (2017), Tariq et al. (2018), Dulina et al. (2019), Bazazan et al. (2019), Moura et al. (2019), and Amin et al. (2020).
Numbness	• Discomfort; unilateral or bilateral loss of sensation.	Schaefer (1982), Hou and Shiao (2006), Kee and Seo (2007), Choobineh et al. (2010), Raithatha and Mishra (2016), and Amin et al. (2020)
Burning feet	• A sensation of feet being uncomfortably hot and painful.	Schaefer (1982), Kee and Seo (2007), Attar (2014), Stolt et al. (2017), and Amin et al. (2020)
Cold feet	• A sensation of feet being uncomfortably cold and painful.	Stolt et al. (2017) and Schaefer (1982)
Bunion, hallux valgus, hallux rigidis, Taylor’s bunion, and bone spurs	• Progressive deformities of foot joints (of the first metatarsophalangeal joint—hallux valgus or the fifth metatarsophalangeal joint—Taylor’s bunion) often occur with a significant functional disability; • Hallux valgus: the first toe visibly turned against the others; • Taylor’s bunion: the fifth toe visibly turned against the others; • Foot pain.	Reed (2007), Nealy et al. (2012), Li (2017), Stolt et al. (2017), and Li, Sommerich, Chipps, Lavender and Stasny (2020)
Hammer toes	• Visible deformities of the foot joints, involving muscles, tendons, and ligaments.	Reed (2007), Nealy et al. (2012), Li (2017), and Stolt et al. (2017)
Low foot arch, high foot arch, flat feet	• Structural congenital or acquired deformities.	Nealy et al. (2012), Stolt et al. (2017), Reed (2007), Li (2017), and Li, Sommerich, Chipps, Lavender and Stasny (2020)

### Reported Prevalence of Foot and Ankle Disorders

The prevalence was significantly heterogeneous between studies on hospital-based contexts (Table 3), with three studies evidencing foot and ankle MSDs as the most prevalent in relation to other body regions. Two studies found that they were the least prevalent.

**Table 3.** Ankle/Foot MSDs (Pain, Numbness, and Tingling) in Hospital-Based Contexts

Authors (year)	12 months	Relation to other body parts
Hou and Shiao (2006)	14.4%	Lp
Kołcz et al. (2019)	16%	Lp
Reed et al. (2014)	55.3%	Third Mp following lower-back and neck problems
Reed (2007)	54%	Third Mp, after lower back (69.9%) and neck problems (66%)
Amin et al. (2020)	47.2%	Mp followed by upper back (40.7%) and shoulders (36.9%)
Almhdawi et al. (2020)	28.5%	First Mp, following lower back (77.4%) and knees (37.5%)
Nguyen et al. (2020)	7.2% (men) 8.8% (women)	Third Lp followed by elbows/forearm (5.9%) and hip/thigh (3.2%)
Stolt et al. (2016)	Ranges between 3.2% and 100%	Mp
Serranheira et al. (2012)	24.83%	First Lp followed by knees (21.74%), thighs (10.51%), and elbows (7.49%)
Nur et al. (2016)	47.2%	Second Mp following neck (48.9%).
Antochevis-de-Oliveira et al. (2017)	41.6%	Fifth Mp following upper back (73.8%), lower back (67.1%), shoulders (52.3%), and neck (42.3%)
Moura et al. (2019)	21.2%	First Lp followed by elbows (14.6%).
Santos et al. (2017)	55.2%	Fourth Lp followed by wrists/hands (51.7%), thighs (34.5%), knees (34.5%), and elbows (24.1%)
Gurgueira et al. (2003)	14.3%	First Lp followed by elbows (7.6%)
Tariq et al. (2018)	35.5%	^a^
Bazazan et al. (2019)	39%	Second Lp followed by elbows (29%).
Sorour and El-Maksoud (2012)	44.8%	^a^
Choobineh et al. (2010)	59%	Second Mp, following lower back (60.6%)
Raithatha and Mishra (2016)	27%	^a^
Yang et al. (2018)	31.5%	^a^
Choobineh et al. (2006)	52.1%	Mp

*Note*. MSD = musculoskeletal disorder; Lp = least prevalent; Mp = most prevalent.

a Prevalence values appear among others, and the classification would be either redundant or irrelevant.

### Walking and Standing Hours

Regarding the hours that nurses spent exposed to standing environments, either walking or in static postures, only five studies (13.16%) reported specific values.

In 1987, Baty and Stubbs conducted an observational study with 52 geriatric nurses, reporting that standing positions accounted for 21.8% of shift time, while walking had 53.7% of shift time. The authors concluded that nurses usually walk more on the late shift—21% of the time, against 18.6% on the early shift—whereas standing is equally distributed throughout the shift—56.6% for the early shift and 53.8% for the late shift.

On the contrary, a study in Brazil (Gonçalves et al., 2001) reported that day shift nurses spend more than 70% of the time standing (roughly 8.4 hours) and that night shift nurses spend about half of the shift (51%) standing (an average of 6.12 hours).

Similar to the previous study, Kołcz et al. (2019) says that 41% of nurses spend more than 8 hours in standing positions and 59% between 5 and 8 hours in these positions.

In a mixed-methods study with 20 nurses from medical/surgical and intensive care unit (ICU) wards, Li, Sommerich, and Chipps (2020) reported 53.7% of shift time was spent standing, 14.7% of shift time walking in ICU wards, and 56.3% of time spent standing and 14.03% of time walking for the latter. For ICU wards, the average time on foot, including time standing and walking, is 68.3%, whereas for medical/surgical wards, the value is slightly higher (70.4%).

More generally, Almhdawi et al. (2020), in a study with 597 nurses, indicated that 44.2% spend long periods in the same position (either static or dynamic), one to three times a day.

### Gender Specifications

In general, female nurses were significantly more affected than male nurses (*p* < .001), reporting MSDs more frequently and presenting a higher prevalence of foot and ankle pain (Nguyen et al., 2020; Omidi et al., 2018 Antochevis-de-Oliveira et al., 2017; Choobineh et al., 2010; Yang et al., 2018). Stolt et al. (2016) considered gender a significant risk factor for developing foot and ankle disorders. Regarding body regions, Stolt et al. (2016) concluded that female nurses have more knee pain (40.4%), whereas male nurses have ankle pain (68.8%) more often.

## Discussion

The recommended practices for safety and health at work provided by the Occupational Safety and Health Administration (OSHA, 2016) suggested that the failure to identify hazards that are either present or could have been anticipated is a root cause for injuries. A root cause analysis recommends the investigation of underlying causes, similar clustering incidents, and common symptomatic trends. Despite this, our findings suggested that nurses’ foot and ankle disorders were still an understudied topic, with no consensus regarding their classifications. Whereas some (Bazazan et al., 2019; Stolt et al., 2015) present a conceptual definition that considers change or damage to the anatomical structures of the ankles and feet (e.g., muscles, tendons, ligaments, and nerves), others offer a functional definition graded in terms of severity (Kee & Seo, 2007). Different conceptual frameworks may have contributed to the heterogeneity of reported symptoms, which undermines the potential for data pooling and comparison. We believe this gap is partly caused by the lack of guidance on what data should be collected to assess foot and ankle disorders, emphasizing the need for an expert-driven consensus that can work as a guideline in future studies.

Although the most reported disorder, none of the included studies comprehensively described pain’s location, radiation, onset mode, character, or intensity. This gap undermines our efforts to highlight current strategies to prevent or mitigate foot and ankle disorders, which could be used to bolster nurses’ health and well-being.

Interestingly, the temporal patterns for nurses’ ankle and foot pain were addressed by some authors (Nur et al., 2016; Stolt et al., 2018), with important implications for occupational health research. After years of exposure to standing environments, especially in hospital-based settings, nurses tend to be more aware and report foot and ankle symptomatology more frequently. Throughout nurses’ professional careers, alongside physiological changes associated with the aging process, standing environments seem to contribute to a gradual, cumulative effect, increasing the severity of the damage caused to local anatomical structures (Amin et al., 2020).

Prevalence rates over 12 months significantly varied between studies, suggesting that nurses’ foot and ankle disorders seem multifactorial in nature, converging with the latest report by the EU-OSHA (2021). Our results showed that organizational factors (e.g., unit layout/design), sociodemographic factors (e.g., age, gender), body mass index (BMI), vascular comorbidities/impairments, specific phenotypes, and self-care behaviors (e.g., type of footwear, lack of physical exercise) contribute to the incidence of foot and ankle disorders.

Despite the risk factors identified in our review, we believe that prevalence rates diverged significantly between authors due to the different conceptual frameworks and the data collection methods used by the authors. For example, in studies where authors used self-reported questionnaires (Amin et al., 2020), prevalence rates were higher than in more objective assessments, as in the case of baropodometric evaluations (Kołcz et al., 2019). This may be due to the subjective nature of the initial symptoms, such as pain and tenderness. Moreover, in earlier stages, common symptoms such as pain may be misinterpreted as an expected outcome after a long working day, disregarding the harmful effects of standing environments over time on nurses’ health and quality of life. Several authors (Antochevis-de-Oliveira et al., 2017; Gurgueira et al., 2003; Santos et al., 2017) identified that nurses perceived foot and ankle disorders as a condition that undermines their well-being and efficiency in the workplace, which, in more severe cases, could lead to absenteeism or desire to change the clinical setting and/or job role.

Our findings must be considered within this review’s limitations. Although not required for scoping reviews, the methodological quality of the included studies was not assessed, which may result in confounding, selection, and information bias. Moreover, research and outcome heterogeneity made it difficult to compare the included studies in specific areas such as the average number of hours spent walking and standing during clinical practice, the type of footwear used and recommended by the healthcare organization (if such recommendations exist), nurses’ previous health comorbidities that can increase the risk of foot and ankle disorders, and studies with samples having more female nurses.

Finally, given that most studies focused on analyzing MSDs of body regions using the Nordic Musculoskeletal Questionnaire, we believe that future studies must focus on developing evidence-informed, robust instruments that identify and classify foot and ankle disorders, standardizing data collection in this field.

## Implications for Occupational Health Nursing Practice

According to the International Council of Nurses (ICN, 2017), the working conditions that nurses are subjected to are among the most dangerous for their health, resulting in occupational injuries, which increase psychological stress and job dissatisfaction. This review synthesized the major foot and ankle disorders, and related signs and symptoms in nurses exposed to standing environments. In addition, it provided insight into prevalence, walking and standing hours, and gender-specific influences on the onset of disorders.

The construction of robust and standardized frameworks for the early identification, prediction, prevention, and personalized treatment of foot and ankle disorders is a good practice for strengthening occupational health policies within organizations. In this sense, current guidelines, recommendations, and position statements on nurses’ occupational health may benefit from the findings of this review. We suggest the following possible directions: (a) cooperation with local occupational health professionals to draw decision algorithms on how to prevent specific disorders as different signs and symptoms require different treatments and preventive measures, including clear recommendations on personalized footwear; (b) raise awareness among healthcare professionals and administrators, actively including them in the characterization of present difficulties and prospective solutions; (c) discuss and endorse occupational strategies that promote nurses’ self-care; (d) promote locally endorsed studies that focus on the prevalence of this health condition among nurses, correlating it with work demands and environmental variables; and (e) international collaboration with professional associations to develop rigorous and evidence-based guidelines for prevention, diagnosis, and treatment of foot and ankle disorders.

**In Summary**

- Nurses are highly exposed to foot and ankle MSDs as they need to walk or stand for long periods. This reality must be adequately identified and described by employers and administrators as professionals might find themselves in a physically stressful situation, leading to absence from work.
- The most common foot and ankle disorder is pain, also called metatarsalgia, generalized forefoot pain, or discomfort. It is an unspecific disorder and should not be confused with the common symptom of “pain” found in most disorders. This elicits the importance of early identification of signs and symptoms and nurses’ awareness of the topic to prevent future incapacitating complications adequately.
- The prevalence of foot and ankle disorders is dependent on a variety of factors, either subjective (awareness level, work-related stress, and level of physical exercise) or objective (nature of work and ward typology, availability of new technology that might alleviate nurses’ work, foot structure, BMI, prior injuries, and footwear). Personalizing occupational health policies to each ward typology and work type is a mandatory intervention.
- Future research is required to provide correlational evidence on the impact of the amount of walking and standing hours on the development of certain foot disorders, how architecture and organizational factors influence nurses’ daily routines and their physical burden, and whether awareness of the topic provides a rapid modification of working habits.

## Supplemental Material

sj-docx-1-whs-10.1177_21650799221137646 – Supplemental material for Foot and Ankle Disorders in Nurses Exposed to Prolonged Standing Environments: A Scoping Review Click here for additional data file. Supplemental material, sj-docx-1-whs-10.1177_21650799221137646 for Foot and Ankle Disorders in Nurses Exposed to Prolonged Standing Environments: A Scoping Review by Rafael A. Bernardes, Sílvia Caldeira, Pedro Parreira, Liliana B. Sousa, João Apóstolo, Inês F. Almeida, Paulo Santos-Costa, Minna Stolt and Arménio Guardado Cruz in Workplace Health & Safety
