# Supplementary material for: Foot and Ankle Disorders in Nurses Exposed to Prolonged Standing Environments: A Scoping Review
Source: Workplace Health Saf. 2023 Jan 13;71(3):101–16. doi: 10.1177/21650799221137646 (PMC9989230; doi:10.1177/21650799221137646)
Supplement: sj-docx-1-whs-10.1177_21650799221137646 – Supplemental material for Foot and Ankle Disorders in Nurses Exposed to Prolonged Standing Environments: A Scoping Review [file sj-docx-1-whs-10.1177_21650799221137646.docx]

**Table 1.**

*Search strings according to each database and number of records found*

*(First search was performed on Medline in December 2020 and the last search on CINAHL Complete in March 2021)*

|  |  | | **Records Retrieved** | | | | | | | | |
| --- | --- | --- | --- | --- | --- | --- | --- | --- | --- | --- | --- |
| **Search** | **Query** | | **Medline (via PubMed)** | **CINAHL Complete (via EBSCOhost)** | **Cochrane** | **SciELO** | | **JBI Connect+, PROSPERO, GoogleScholar, OpenGrey, OpenDOAR, ProQuest Dissertations** | | | |
| #1 | ((((((nurs*[Title/Abstract]) OR (“nurse practitioner”[Title/Abstract])) OR  (“nurse practitioners”[Title/Abstract])) OR (“nursing  personnel”[Title/Abstract])) OR (“registered nurses”[Title/Abstract])) OR  (“registered nurse”[Title/Abstract])) OR (nurse[MeSH Terms]) | | 436,175 | 234,543 | 54 | 2,100 | |  |  | 0 |  |
| #2 | (“foot disorders”[Title/Abstract] OR “foot health”[Title/Abstract] OR “foot  diseases”[Title/Abstract] OR “foot disease”[Title/Abstract] OR “foot  diseases”[MeSH Terms]) | | 17,648 | 2,323 | 123 | 156 | |  |  | 0 |  |
| #3 | (“standing”[Title/Abstract] OR “long standing”[Title/Abstract] OR  “prolonged standing”[Title/Abstract] OR “prolonged walking”[Title/Abstract]  OR “standing position”[Title/Abstract] OR “standing  positions”[Title/Abstract]) | | 75,039 | 54 | 6 | 67 | |  |  | 0 |  |
| #4 | #1 OR #2 | | 453,583 | 236,866 | 170 | 2,254 | |  |  | 0 |  |
| #5 | #2 OR #3 | | 92,458 | 23 | 177 | 66 | |  |  | 0 |  |
| #6 | #1 OR #3 | | 509,912 | 12 | 3 | 2.200 | |  |  | 0 |  |
| #7 | #4 AND #5 AND #6 | | 1,761 | 231 | 23 | 123 | |  |  | 0 |  |
| **Records Retrieved from WoS*** | | | | | | | | | | | |
| **Search** | | **Query** | | | | | **WoS** | | | | |
| #1 | | (T/A(“nurse”) OR T/A(“nurses”) OR T/A (registered nurses”)) | | | | | 5,566 | | | | |
| #2 | | (T/A(“foot disease”) OR T/A(“feet disease”)) | | | | | 28 | | | | |
| #3 | | (T/A(“stand”) OR (“standing”) OR (“static position”)) | | | | | 7 | | | | |
| #4 | | #1 OR #2 | | | | | 5846 | | | | |
| #5 | | #2 OR #3 | | | | | 39 | | | | |
| #6 | | #1 OR #3 | | | | | 5,590 | | | | |
| #7 | | #4 AND #5 AND #6 | | | | | 46 | | | | |

**CINAHL:** Cumulative Index to Nursing and Allied Health Literature; **WoS:** Web of Science; **Filters:** Portuguese, English, French and Spanish studies, ranging from 1970 onwards; *Search on WoS didn’t not use MeSH Terms
